# Validation of *Myc*-Associated Protein X (MAX) regulation in growth hormone secreting and nonfunctional pituitary adenoma

**DOI:** 10.1371/journal.pone.0284949

**Published:** 2023-04-27

**Authors:** Douglass W. Tucker, Dhiraj J. Pangal, Robin Du, Angad S. Gogia, Ali Tafreshi, Jacob Ruzevick, Kyle T. Hurth, Tim Triche, Alexander Micko, John D. Carpten, Mark S. Shiroishi, John D. Carmichael, Suhn K. Rhie, Gabriel Zada

**Affiliations:** 1 USC Brain Tumor Center, Department of Neurosurgery, Keck School of Medicine of the University of Southern California, Los Angeles, California, United States of America; 2 Department of Clinical Pathology, Keck School of Medicine of the University of Southern California, Los Angeles, California, United States of America; 3 Center for Epigenetics, Van Andel Research Institute, Grand Rapids, Michigan, United States of America; 4 Department of Neurosurgery, Medical University of Vienna, Vienna, Austria; 5 Department of Translational Genomics, Keck School of Medicine of the University of Southern California, Los Angeles, California, United States of America; 6 Department of Radiology, Keck School of Medicine of the University of Southern California, Los Angeles, California, United States of America; 7 Department of Endocrinology, Keck School of Medicine of the University of Southern California, Los Angeles, California, United States of America; 8 Department of Biochemistry and Molecular Medicine, Keck School of Medicine of the University of Southern California, Los Angeles, California, United States of America; farhat hached university hospital Republic of Tunisia Ministry of Public Health: Republique Tunisienne Ministere de la Sante Publique, TUNISIA

## Abstract

**Introduction:**

Many patients with growth hormone-secreting pituitary adenoma (GHPA) fail to achieve biochemical remission, warranting investigation into epigenetic and molecular signatures associated with tumorigenesis and hormonal secretion. Prior work exploring the DNA methylome showed *Myc*-Associated Protein X (MAX), a transcription factor involved in cell cycle regulation, was differentially methylated between GHPA and nonfunctional pituitary adenoma (NFPA). We aimed to validate the differential DNA methylation and related MAX protein expression profiles between NFPA and GHPA.

**Methods:**

DNA methylation levels were measured in 52 surgically resected tumors (37 NFPA, 15 GHPA) at ~100,000 known MAX binding sites derived using ChIP-seq analysis from ENCODE. Findings were correlated with MAX protein expression using a constructed tissue microarray (TMA). Gene ontology analysis was performed to explore downstream genetic and signaling pathways regulated by MAX.

**Results:**

GHPA had more hypomethylation events across all known MAX binding sites. Of binding sites defined using ChIP-seq analysis, 1,551 sites had significantly different methylation patterns between the two cohorts; 432 occurred near promoter regions potentially regulated by MAX, including promoters of TNF and MMP9. Gene ontology analysis suggested enrichment in genes involved in oxygen response, immune system regulation, and cell proliferation. Thirteen MAX binding sites were within coding regions of genes. GHPA demonstrated significantly increased expression of MAX protein compared to NFPA.

**Conclusion:**

GHPA have significantly different DNA methylation and downstream protein expression levels of MAX compared to NFPA. These differences may influence mechanisms involved with cellular proliferation, tumor invasion and hormonal secretion.

## Introduction

Growth hormone-secreting pituitary adenoma (GHPA) cause significant clinical morbidity and mortality [1, 2]. Despite advances in surgical technique and improvements in adjuvant medical and radiosurgical therapies, between 20% and 40% of patients with GHPA fail to achieve hormonal remission [3–5]. Nonfunctional pituitary adenoma (NFPA) portend a more favorable outcome for patients but are the most common pituitary adenoma (PA) subtype. Because these tumors often present following mass effect related symptoms, tumors have often grown to encompass the carotid arteries or extend into the extrasellar spaces, thus making them unamenable to complete surgical resection [6, 7]. Understanding the biochemical pathways and epigenetic modifications which govern GHPA and NFPA tumorigenesis and hormone secretion (for GHPA) is an understudied problem with clear clinical applications for patients with PA. However, in the literature, few mutations or pathways driving tumorigenesis have been identified [8, 9].

Epigenetic changes have been implicated in cancer and the loss of cell growth control in a variety of tumors, including PA [10, 11]. Our team previously showed that DNA hypermethylation of the *KCNAB2* promoter region is found in nonfunctional pituitary adenoma (NFPA) [10, 12]. Transcription factors, which alter expression of numerous genes by binding regulatory elements such as promoters and enhancers, are a growing area of interest within PA research and diagnostics [13]. Epigenetic modifications that contribute to GHPA tumorigenesis or drive tumor progression, particularly with regards to transcription factor activity, are largely unstudied.

The transcription factor *Myc* Associated Factor X (MAX) is a key component of cell cycle regulation. *MAX* mutations have been implicated in PA tumorigenesis as well as other neurological malignancies [14–17]. A prior exploratory analysis of the DNA methylome by our team showed that *MAX* was widely differentially methylated between various subtypes of PA, particularly GHPA and NFPA [10]. Despite these studies indicating that *MAX* may be implicated in neuroendocrine tumors, the role of *MAX* in PA biological behavior has not been validated.

To further classify the role of *MAX* modification in GHPA and how that may differ from NFPA, we investigated DNA methylation patterns of *MAX* between surgically resected GHPA and NFPA. We then correlated these findings with MAX protein expression between the two cohorts and finally performed a gene ontology analysis to explore downstream genetic and signaling pathways regulated by *MAX*.

## Materials and methods

Pathologic specimens from fifty-two patients (37 NFPA, 15 GHPA) who underwent endoscopic endonasal resection of histologically-validated PA at the Keck Hospital of USC by a single surgeon were obtained [10]. Written informed consent protocol as outlined by our institution was followed and the study was approved by the Institutional Review Board of the University of Southern California. Following acquisition of the specimen, the following analyses were performed: DNA methylation analysis and immunohistochemical tissue microarray (TMA) development. Downstream gene characterization and statistical analyses were then performed using the results of these studies. These methods are outlined in detail below.

### DNA methylation analysis

PA samples were bisulfite converted using the Zymo EZ DNA Methylation kit (Zymo Research, Irvine, CA), and DNA methylation levels were profiled using the Illumina Infinium HumanMethylation (HM450) Beadchip array in the USC Epigenome Center. Transcription factor binding sites for MAX were generated from 91 samples using the ENCODE database (n = 103,739 binding sites) [18]. DNA methylation levels were normalized, and values under 0.7 were considered hypomethylation events based on methodologies used by our institutional core laboratory and prior published cutoffs [10, 19].

### Gene analysis

Genes with MAX binding sites within 2kb of transcription start sites (TSS) as defined by GENCODE were identified as those who contain MAX binding sites in their promoter region [20, 21]. Using RCircos package, the location of these genes was plotted [22]. These sites are hypomethylated sites in GHPA compared to NFPA divided by all MAX binding sites, defined using ENCODE ChIP-seq data. Gene ontology analysis was performed with the identified genes using Gene Set Enrichment Analysis (GSEA) MsigDB compute overlaps tools, and the top 10 gene ontology categories ranked by–log(false discovery rate) were plotted (Fig 2B).

### Immunohistochemistry

Following IRB approval for construction, 3 mm sections were obtained from our surgical tumor bank (GH = 23, NFPA = 39, normal pituitary = 3). Normal pituitary gland obtained from cadaveric specimens who died from non-pituitary related neurodegenerative disease was used as a negative control. This data is provided in S1 Fig. For TMA construction, 2mm diameter patient pituitary tumor specimens were placed on a charged slide and refrigerated within 24 hours of sectioning. Slides were air dried and stored at room temperature for the duration of the study. Slides were baked in 60°C oven for 60 minutes. Slides were stained for IHC using the anti-MAX antibody (Abcam, ab101271) on the Leica Bond III Automated Stainer (Leica Biosystems, Buffalo Grove, IL). A positive batch tissue control, sectioned at 4μm, was included with each staining run, for a total of 2 staining runs. Protocols were provided by clinical USC IHC lab. Primary antibody was MAX from AbCam (ab101271) at a 1:500 dilution. Scoring criteria was defined as per Tucker et al. in 2018, where scores for strength of positivity (0–3) and the percentage of cells positive (0 = <1%, 1 = 1–10%, 2 = 10–25%, 3 = 25–75%, 4 = >75%) were added [23].

### Statistical analysis

Statistical analyses were performed using GraphPad Prism 8 software (GraphPad Software, La Jolla, California, USA). Significance between independent datasets was determined using Student T-Test for continuous data and chi-square tests for categorical data. Results were deemed statistically significant if p<0.05. Methylation analyses were performed using R; implementation details for the purpose of reproducibility are shown in the S1 Appendix.

## Results

### ENCODE derived MAX binding sites are hypomethylated in GHPA

**Fig 1:**
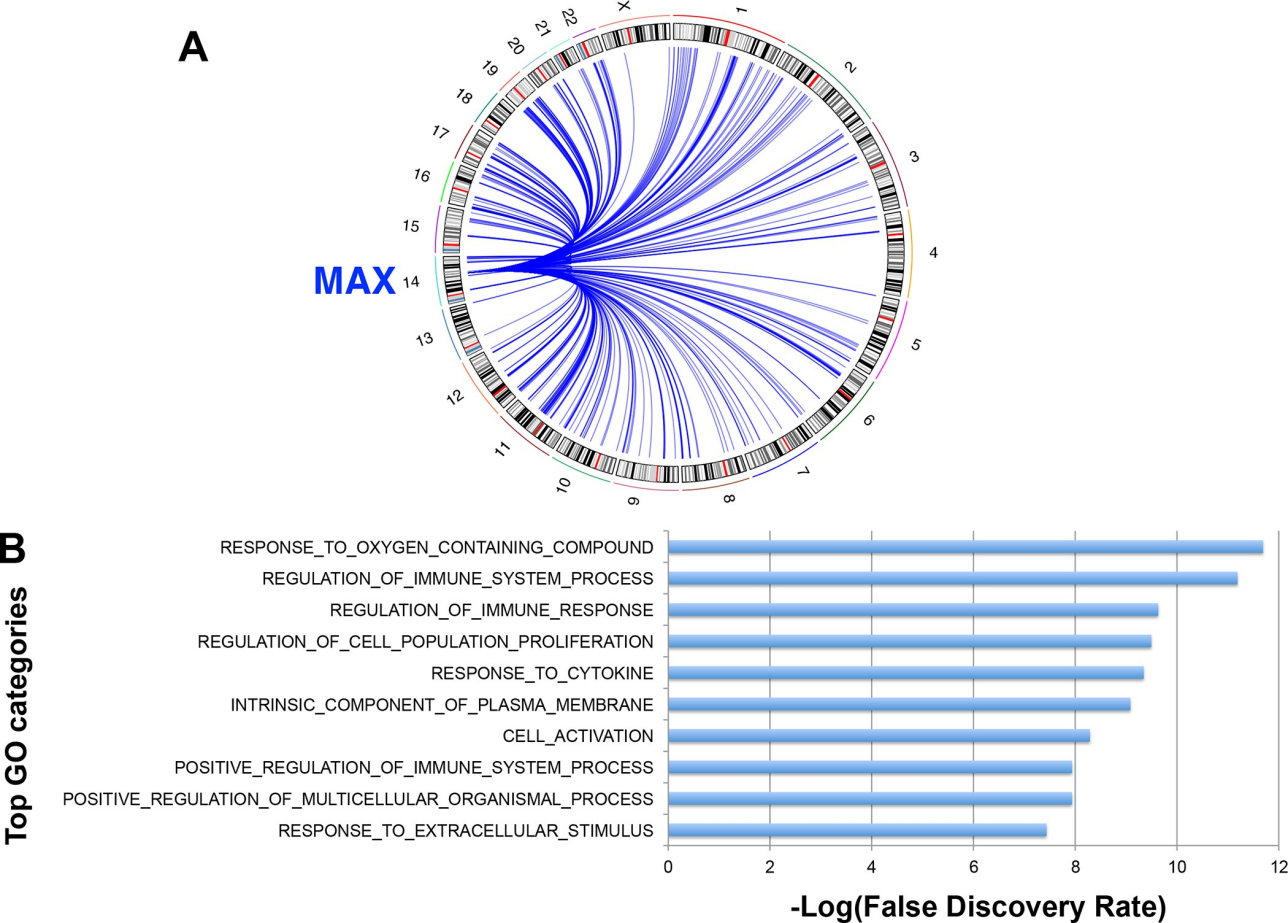
Genes with differentially methylated MAX binding sites at their promoters. **(A)** Shown is a Circos plot showing the location of genes with differentially methylated MAX binding sites. **(B)** Bar plot showing top gene ontology categories of differentially methylated MAX binding sites.

By incorporating MAX ChIP-seq data from ENCODE, we identified 103,739 total transcription binding sites for analysis. Epigenetic changes in binding sites of MAX were measured by DNA methylation levels at these sites, and we found GHPA are globally hypomethylated compared to NFPA (Fig 1A). In addition, 1551 specific binding sites were significantly (p<0.05) hypomethylated in GHPA than NFPA (Fig 1B) [21]. Of these differentially methylated MAX binding sites, 13 could be mapped to specific genes (Table 1).

**Table 1:** Differentially methylated MAX binding sites between NFPA vs. GHPA.

probeID	geneID	distToTSS	p-value
cg22171607	RP11-431K24.1	50765	0.038554136
cg24661860	CASZ1	72343	0.029309093
cg11348165	SFN	-360	0.017538046
cg22356428	RASSF5	6142	0.01056245
cg16745930	HPSE2	283020	0.001638185
cg04737124	VTI1A	137970	0.038140607
cg02793828	LOC400548	25360	0.001497124
cg17864646	SECTM1	-5228	0.016084048
cg07058988	SECTM1	-5238	0.023824799
cg21559943	PTPRH	-415	0.007166712
cg02686793	WISP3	34944	0.040263505
cg11006453	AGO2	16803	0.009608558
cg14458315	GABBR2	392173	0.038619428

### Genes potentially regulated by MAX in GHPA

**Fig 2:**
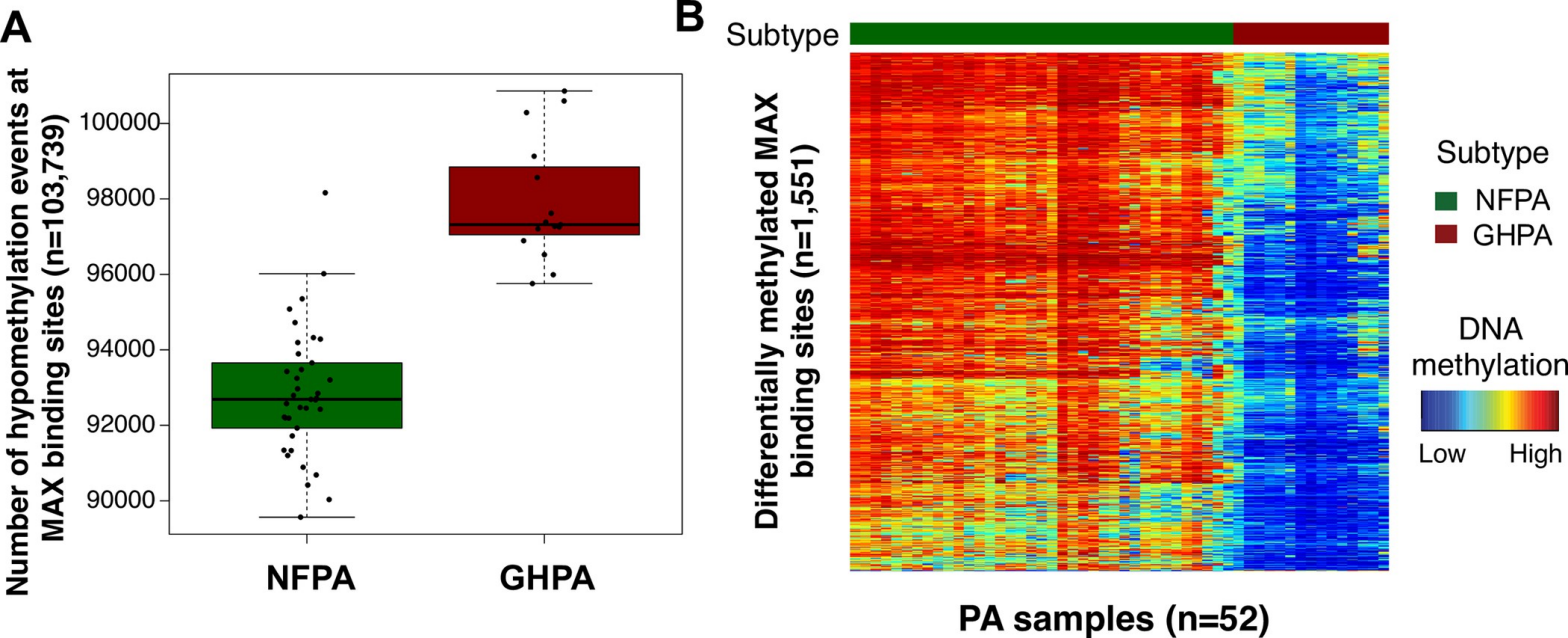
MAX binding sites are hypomethylated in GH-secreting PA compared to Non-functional PA. **(A)** Number of hypomethylated MAX binding sites in NFPA (n = 37) vs GHPA (n = 15) (p<1.22e-10). **(B)** Heat map displaying loci of differentially methylated MAX binding sites between NFPA and GHPA (p<0.05) (n = 13 probes).

We identified 432 genes with promoter regions demonstrating differentially hypomethylated MAX binding sites (Fig 2A) (S1 Appendix) [21]. Gene ontology categories most enriched as a result of the differentially methylated sites included: response to oxygen containing compounds, immune regulation, and cell proliferation (Fig 2B).

### MAX expression is increased in GHPA compared to NFPA

**Fig 3:**
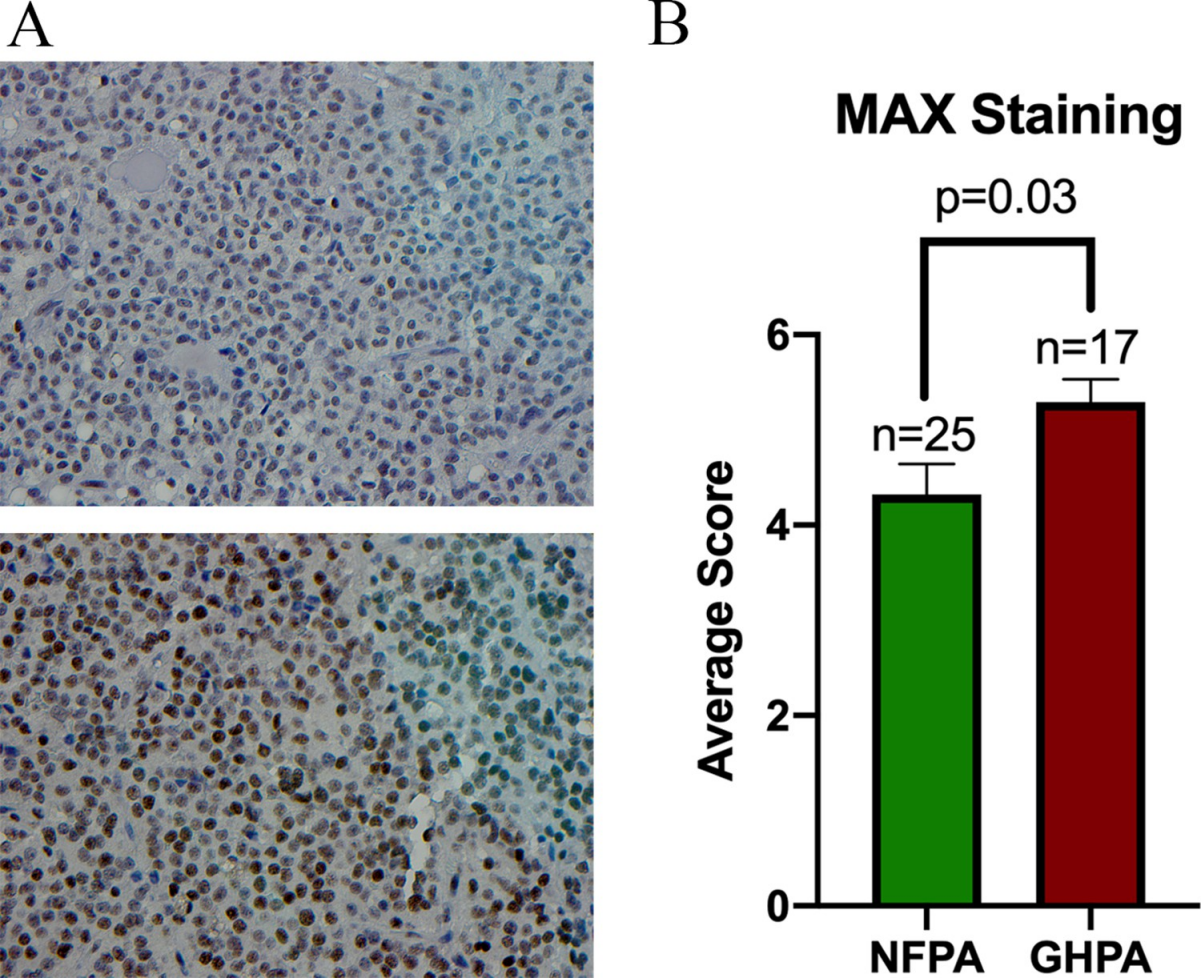
MAX is overexpressed in GH-secreting PA compared to Non-functional PA. **(A)**Representative images of MAX protein expression levels for GHPA and NFPA tissue utilizing IHC. **(B)** MAX protein expression is significantly increased in GHPA when compared to NFPA (average score of 9.2 ± 4.0 vs 7.0 ± 2.8, p = 0.02).

We additionally performed an independent immunohistochemistry analysis on a previously constructed tissue microarray of surgically resected patient samples of both NFPA and GHPA. GHPA and NFPA cohort TMAs were stained for MAX protein expression (Fig 3A). Notably, there was increased protein expression of MAX in the GHPA (n = 23) compared to the NFPA (n = 39) cohort (9.2 ± 4.0 vs 7.0 ± 2.8, p = 0.02) (Fig 3B).

## Discussion

Acromegaly caused by GHPA remains a challenging pathology with significant clinical implications, particularly for patients who fail to achieve hormonal remission following aggressive surgical, radiosurgical, and/or pharmacologic therapy [1, 2, 4]. In this study, we confirm a novel finding of differential epigenetic modification of MAX binding sites and associated protein expression of MAX between GHPA and NFPA. We further describe potential related effects on downstream biological activity based on gene ontology analysis.

### Role of MAX

The 2017 edition of the World Health Organization (WHO) classification of endocrine tumors emphasized lineage-specific pituitary transcription factors to classify pituitary adenoma, specifically PIT1 (pituitary specific transcription factor 1) and TPIT (pituitary cell restricted factor) [13, 24]. Given this emphasis, investigation into other adenoma subtype specific transcription factors is warranted and may be indicative of clinical characteristics such as tumor aggression or possibility of recurrence.

The oncogenic transcription factor MAX is a key component of the cell cycle. MAX forms homodimers and heterodimers with other transcription factors such as MYC, MXI1, MNT, and MXD1. Multiple reports have highlighted the involvement of *MAX* and *MYC* genes in PA. For example, germline *MAX* mutations are associated with neuroendocrine tumors including prolactinomas and pheochromocytomas [14, 15, 17]. Additionally, *c-MYC* was overexpressed in pituitary tumors that were classified as aggressive, larger in size, and diagnosed at a younger age [25, 26]. No prior studies, however, have validated differences in the MYC/MAX pathway when comparing NFPA and GHPA subtypes.

### MAX hypomethylation and increased gene expression

DNA methylation is an epigenetic mechanism by which chromatin is made more accessible or less accessible to transcription (hyper, hypo-methylation, respectively). In this study, we show that MAX transcription factor binding sites are globally hypomethylated and demonstrate increased accessibility for transcription factor binding in GHPA compared to NFPA. Using an independent dataset, we also corroborated that MAX protein expression levels are elevated in GHPA compared to NFPA, perhaps working as a component of a positive feedback loop. These results also agree with the findings of Salomon et al., who reported differing methylation profiles between PA subtypes [11]. More directly, Garcia-Martinez et al., demonstrated that *myc* was differentially expressed in somatotroph adenoma compared to gonadotroph adenoma, and that degree of tumor invasion was correlated with degree of *myc* expression [27]. Our study complements these results, as hypomethylation events represent an amplification of the downstream effects of the *MAX* transcription factor. Nonetheless, these authors urge further validation of possible biomarkers and mechanisms for tumor invasion and subtype specific growth.

As transcription factors often act on promoter regions to regulate gene expression, we investigated which hypomethylated MAX binding sites were in promoter regions; in total 432 were identified. Secondary analysis using gene ontology demonstrated that these genes regulated by MAX binding sites are involved in responses to oxygen containing compounds, immune regulation, and cell proliferation. Target genes of MAX in GHPA included known genes involved in pituitary tumorigenesis. For example, tumor necrosis factor (TNF) has been shown to have direct effects on cultured anterior pituitary cells, blunting the release of hormones in response to hypothalamic factors [28]. MMP9, whose activation is correlated with invasion of pituitary null cell adenoma, has a hypomethylated promoter with MAX binding in GHPA [29]. DUSP1 is known to be involved in thyroid hormone-mediated apoptosis and finally the gene SFN has been shown to have a hypermethylated promoter region in NFPA compared to normal pituitary samples [30, 31]. A list of identified genes (S1 Appendix) as well as identified molecular mechanisms involving MAX may provide new insights into understanding the biological behavior of PA subtypes is shown.

### Future directions

Standard treatment for GHPA which fail surgical or radiosurgical therapy includes somatostatin receptor therapy (SSRT), which activates a cellular pathway that ultimately inhibits GH secretion. These medications (most commonly octreotide and lanreotide) have limited effectiveness, as up to 10% of all patients have an innate resistance to medical therapy, and long-term response is seen in only 17–41% of cases [32–35]. Other medical therapies such as pegvisomant (GH receptor antagonist) and cabergoline (dopamine agonist) improve IGF-1 in a subset of patients with medication resistant acromegaly, but rates of biochemical non-remission range from 25–40% in this cohort [36–38]. Due to this high failure rate, novel therapeutic approaches are needed, especially for patients who show resistance or are poor candidates for additional surgical management. Castell et al. in 2018 reported a novel MYC:MAX dimerization inhibitor which was able to inhibit tumor growth and induce apoptosis in both in vitro and a xenograft model of neuroblastoma, a finding worth studying in PA given our results [39].

### Limitations

There are several limitations to the current study which coach our findings in exploratory analysis which warrant further study. Though we describe clear differences in areas of hypomethylation and MAX protein expression between GHPA and NFPA the specific binding of these transcription factors was not confirmed using CHIP-seq data. To further validate and confirm these findings it is necessary to utilize other confirmatory methods, including CHIP-seq or pyrosequencing, in a new cohort of data. It would be worthwhile to find patients whom preserving normal pituitary gland during adenoma resection is not possible, which by then an internal control of normal gland would be obtained. In addition, we lack tissue culture proliferation assays showing differential aggressiveness in between NFPA and GHPA, though we plan this for future studies. Finally, our gene ontology studies are preliminary and merely demonstrate the potential for downstream, tissue and organ system-based effects to be seen by changes to the cellular or epigenetic milieu. Despite these limitations, we believe our work demonstrates the potential for further biological study into the epigenetic modifications of MAX binding sites as a potential therapeutic target.

## Conclusions

In this study, we validate molecular and epigenetic difference between NFPA and GHPA with clear implications to downstream molecular pathways known to be involved in tumorigenesis. Further studies are warranted to assess the role of targeted epigenetic and molecular therapeutics for GHPA.

## Supporting information

S1 Fig Differentially methylated MAX binding sites between NFPA vs. GHPA. (TIFF)

S1 Appendix Details regarding DNA methylation methods, table showing additional DNA promoter sites. (DOCX)
