# Supplementary figures and images for: Validation of Myc-Associated Protein X (MAX) regulation in growth hormone secreting and nonfunctional pituitary adenoma
Source: PLoS One. 2023 Apr 27;18(4):e0284949. doi: 10.1371/journal.pone.0284949 (PMC10138191; doi:10.1371/journal.pone.0284949)

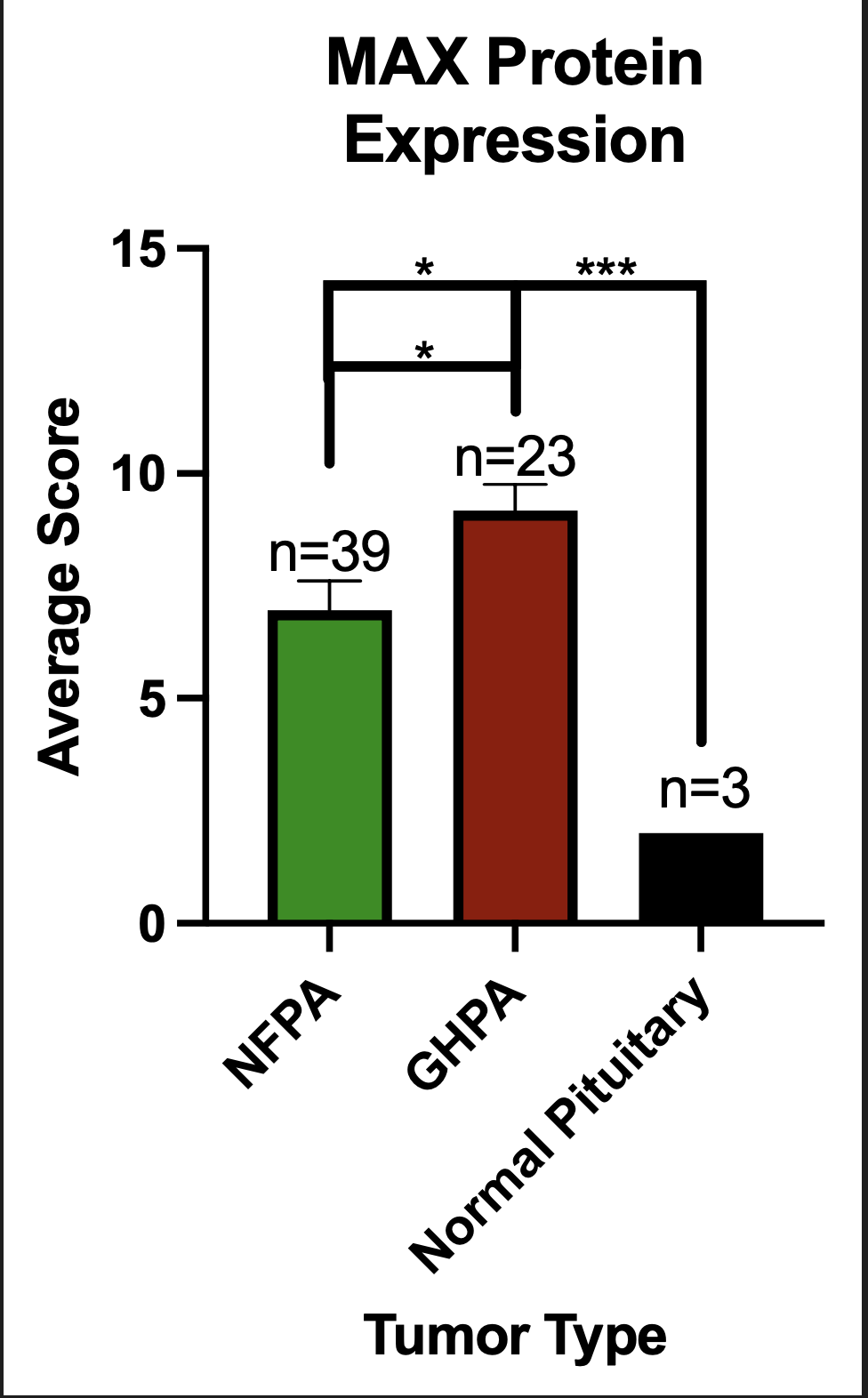

Supplement: S1 Fig — (TIFF) [file pone.0284949.s001.tiff]
