# Supplementary material for: Validation of Myc-Associated Protein X (MAX) regulation in growth hormone secreting and nonfunctional pituitary adenoma
Source: PLoS One. 2023 Apr 27;18(4):e0284949. doi: 10.1371/journal.pone.0284949 (PMC10138191; doi:10.1371/journal.pone.0284949)
Supplement: S1 Appendix — (DOCX) [file pone.0284949.s002.docx]

Appendix

*DNA Methylation*

PA samples were bisulfite converted using the Zymo EZ DNA Methylation kit (Zymo Research, Irvine, CA), and DNA methylation levels were profiled using the Illumina Infinium HumanMethylation (HM450) Beadchip array in the USC Epigenome Center. Normalized DNA methylation levels (betas) were calculated by processing raw IDAT files using miser R package (https://github.com/ttriche/miser). Transcription factor binding sites generated from 91 samples from ENCODE[[14]](https://paperpile.com/c/4YOxyO/CHjsv) were downloaded (<https://zwdzwd.github.io/InfiniumAnnotation/>) and HM450 probes located at MAX binding sites were selected (n=103,739). Hypomethylation events were calculated using betas cut off 0.7 for each probe per sample and the number of hypomethylation events at MAX binding sites were plotted in R (https://www.r-project.org/) for Figure 1A. Differentially methylated MAX binding sites between NFPAs and GHPAs were identified using sesame R package (<https://bioconductor.org/packages/release/bioc/html/sesame.html>)[[15]](https://paperpile.com/c/4YOxyO/oCSoy) (p<0.05) (Supplementary Table 1) and plotted as a heatmap in R (<https://www.r-project.org/>) (Figure 1B).

**Supplemental Table 1**

A list of genes that have hypomethylated MAX binding sites at their promoters in GHPAs compared to NFPAs.

| PLEKHN1 | POU6F1 | BCOR | NOL12 | MIR4669 | CA7 |
| --- | --- | --- | --- | --- | --- |
| TAS1R3 | SMAGP | LCA10 | BAIAP2L2 | CARD9 | RRAD |
| NPPA-AS1 | KRT80 | AC067852.1 | RP3-370M22.8 | LCN8 | CES3 |
| NPPB | KRT86 | WNK4 | FAM83F | SLC34A3 | DPEP2 |
| KLHDC7A | RP3-416H24.1 | SOST | PARVG | CACNA1C-AS1 | HSD17B2 |
| GALE | RP11-686F15.3 | RP11-304F15.4 | IL17RE | FGF23 | COTL1 |
| SFN | HOXC4 | SPATA20 | SLC6A11 | LTBR | RP4-536B24.4 |
| RP11-73M7.1 | NCKAP1L | LINC00483 | RP11-193I22.2 | SCNN1A | CYBA |
| RP11-268J15.5 | RBMS1P1 | ERN1 | COL7A1 | LPAR5 | AC137932.6 |
| EDN2 | AC078889.1 | ARSG | GNAT1 | C3AR1 | RP11-1260E13.2 |
| ARTN | MIR5700 | EVPL | SEMA3B | RP11-20D14.3 | RP11-1260E13.3 |
| FAM159A | Y_RNA | ST6GALNAC1 | SEMA3B-AS1 | MFAP5 | MYBBP1A |
| PCSK9 | HNF1A | FLJ45079 | RP11-168J18.6 | PLBD1 | GGT6 |
| RP11-145M4.3 | P2RX7 | RP11-149I9.2 | SEMA3G | RP11-695J4.2 | TM4SF5 |
| RP11-145M4.1 | KDM2B | MIR1250 | SMIM4 | C1QL4 | RP5-1050D4.3 |
| CHI3L2 | RP13-941N14.1 | RP11-498C9.2 | NT5DC2 | CD68 | SPAG7 |
| MIR942 | RP11-463O12.3 | RP11-498C9.3 | ADAMTS9-AS2 | RP11-599B13.3 | ASGR2 |
| HFE2 | LRCOL1 | NOTUM | RP11-326J18.1 | TNFRSF13B | ASGR1 |
| ECM1 | RPS21P8 | DUS1L | CD80 | EVPLL | FLJ14816 |
| S100A3 | F7 | RNU6-1223P | RP11-158I23.1 | MFAP4 | WNT6 |
| S100A4 | F10 | C19orf77 | LINC00881 | ARL5C | AC106876.2 |
| S100A2 | TRAV36DV7 | AC004637.1 | FAM131A | RP5-906A24.2 | NGEF |
| IL6R | TRAJ33 | PLIN4 | THPO | GRB7 | INPP5D |
| RP11-350G8.5 | TRAJ34 | SEMA6B | CHRD | RP11-387H17.6 | AC096574.5 |
| LENEP | TRAJ32 | DENND1C | IDUA | CSF3 | KLHL30 |
| MUC1 | TRAJ31 | FCER2 | DGKQ | KRT15 | AGXT |
| PKLR | AL135998.1 | ANGPTL6 | RNU6-204P | MX2 | BPIFB2 |
| RP11-284F21.7 | CEBPE | P2RY11 | MIR4274 | KRTAP10-6 | SLA2 |
| RP11-66D17.3 | RP11-517O13.3 | MIR5684 | TLR6 | SLC25A18 | TLDC2 |
| SH2D2A | TOMM20L | CTD-2659N19.9 | TLR1 | MIR4761 | SRC |
| NTRK1 | MAX | KLF1 | TMEM156 | MIR1306 | TGM2 |
| SLAMF8 | ZFP36L1 | MIR181C | RP11-395I6.2 | MIR3618 | SLPI |
| FCGR2A | CTD-2547L24.3 | RLN3 | LIMCH1 | MIR130B | MMP9 |
| RP11-408E1.1 | RP11-895M11.3 | CYP4F12 | SLC12A7 | MIR301B | OCSTAMP |
| DENND1B | CCDC88C | CTD-2278I10.4 | CTD-3080P12.3 | PPIL2 | LAMA5 |
| RP11-510N19.5 | ASB2 | CTD-2521M24.9 | CTC-537E7.3 | MTFP1 | RP4-583P15.14 |
| RP11-465N4.4 | MIR345 | BST2 | RP11-229C3.4 | AL049747.1 | LIME1 |
| PTPN7 | SLC25A29 | MVB12A | PDLIM4 | MROH6 | RP13-152O15.5 |
| IL24 | CTD-2555C10.3 | CTD-2527I21.4 | CTC-349C3.2 | TPD52L3 | IFNGR2 |
| CAPN2 | RP11-982M15.7 | AC020907.2 | SNORA74 | AQP3 | BRWD1-AS1 |
| GJC2 | RP11-982M15.6 | FXYD1 | GPR151 | RP11-311H10.7 | IFRD1 |
| C1orf100 | INF2 | ZBTB32 | MIR145 | CA9 | LSMEM1 |
| LYPD8 | RP11-521B24.3 | TYROBP | MIR143 | ARHGEF39 | RP11-128A6.3 |
| AKR1C2 | MTA1 | CD79A | DUSP1 | HRCT1 | KEL |
| FXYD4 | PLCB2 | TMEM145 | CANX | TJP2 | ATG9B |
| HNRNPA1P33 | LTK | ZNF296 | HMGB3P22 | RP11-49O14.2 | FASTK |
| HMGN2P34 | TYRO3 | SIX5 | CBY3 | NR5A1 | TMUB1 |
| ANKRD1 | TCEB1P2 | AC074212.6 | CTC-205M6.5 | SH2D3C | MIR3926-2 |
| RNU6-740P | EPB42 | PPP1R15A | RP11-288G3.3 | FOSL1 | RP11-380I10.4 |
| RP11-310E22.4 | PSTPIP1 | CTB-60B18.10 | SLC17A2 | CLCF1 | STAR |
| LZTS2 | RP11-114H24.6 | HRC | ZSCAN31 | AP003419.11 | KB-1410C5.3 |
| RP11-108L7.15 | MESDC1 | TRPM4 | TRIM27 | PITPNM1 | CTHRC1 |
| KAZALD1 | FSD2 | CD37 | PSORS1C1 | ALDH3B1 | U3 |
| PRLHR | ALPK3 | CLEC11A | PSORS1C3 | LRP5 | RP11-539E17.5 |
| INPP5A | CTD-2262B20.1 | SIGLEC17P | LTA | MRGPRF | KLHL38 |
| IFITM1 | SPATA41 | PTPRH | TNF | RP11-554A11.6 | FER1L6-AS1 |
| RP11-496I9.1 | MPG | TMEM190 | LST1 | RP11-169D4.1 | RP11-959I15.4 |
| MRPL23-AS1 | RHBDF1 | CTD-2105E13.15 | CSNK2B | RP11-169D4.2 | PTP4A3 |
| AC051649.6 | RAB11FIP3 | COLEC11 | CFB | RP11-800A3.4 | CTD-3064M3.4 |
| SLC22A18 | WDR90 | ALLC | STK19 | AP001324.1 | JRK |
| PHLDA2 | AL022341.3 | AC092687.3 | HSD17B8 | TSKU | LY6E |
| PRKCDBP | RP11-616M22.7 | AC023137.2 | B3GALT4 | RP11-21L23.3 | RP11-661A12.9 |
| LDHA | PTX4 | UCN | GGNBP1 | RAB30-AS1 | SPDYC |
| RP11-613D13.8 | RP11-304L19.8 | GCKR | TREML2 | RAB30 | SIPA1 |
| CTD-2560E9.5 | BRICD5 | FNDC4 | NCR2 | RP11-708L7.6 | SOCS1 |
| CTD-2589M5.4 | HCFC1R1 | LBH | CEP57L1 | TMPRSS4 | RP11-21M24.2 |
| CHRM4 | THOC6 | AC007365.3 | SESN1 | PHLDB1 | FHL2 |
| PRG3 | CCDC64B | DYSF | RP11-732M18.2 | RP11-158I9.5 | AC016683.5 |
| PTGDR2 | IL32 | ATOH8 | RP3-497J21.1 | MIR4492 | DDC |
| RAB3IL1 | SEPT12 | AC012307.2 | RP11-302L19.3 | NINJ2 | RHBDD2 |
| CHRM1 | SMIM22 | ZAP70 | AC147651.4 | SULT1A2 | ITGAL |
| LGALS12 | CIITA | IL1R1 | CYP2W1 | CD19 | PRSS8 |
